# Insulin Regulates the Activity of the High-Affinity Choline Transporter CHT

**DOI:** 10.1371/journal.pone.0132934

**Published:** 2015-07-10

**Authors:** Katherine J. Fishwick, R. Jane Rylett

**Affiliations:** 1 Molecular Medicine Research Group, Robarts Research Institute, Schulich School of Medicine & Dentistry, University of Western Ontario, London, Ontario, Canada; 2 Department of Physiology and Pharmacology, Schulich School of Medicine & Dentistry, University of Western Ontario, London, Ontario, Canada; Tohoku University, JAPAN

## Abstract

Studies in humans and animal models show that neuronal insulin resistance increases the risk of developing Alzheimer’s Disease (AD), and that insulin treatment may promote memory function. Cholinergic neurons play a critical role in cognitive and attentional processing and their dysfunction early in AD pathology may promote the progression of AD pathology. Synthesis and release of the neurotransmitter acetylcholine (ACh) is closely linked to the activity of the high-affinity choline transporter protein (CHT), but the impact of insulin receptor signaling and neuronal insulin resistance on these aspects of cholinergic function are unknown. In this study, we used differentiated SH-SY5Y cells stably-expressing CHT proteins to study the effect of insulin signaling on CHT activity and function. We find that choline uptake activity measured after acute addition of 20 nM insulin is significantly lower in cells that were grown for 24 h in media containing insulin compared to cells grown in the absence of insulin. This coincides with loss of ability to increase phospho-Protein Kinase B (PKB)/Akt levels in response to acute insulin stimulation in the chronic insulin-treated cells. Inhibition of phosphatidylinositol-4,5-bisphosphate 3-kinase (PI3-kinase) in cells significantly lowers phospho-PKB/Akt levels and decreases choline uptake activity. We show total internal reflection microscopy (TIRF) imaging of the dynamic movement of CHT proteins in live cells in response to depolarization and drug treatments. These data show that acute exposure of depolarized cells to insulin is coupled to transiently increased levels of CHT proteins at the cell surface, and that this is attenuated by chronic insulin exposure. Moreover, prolonged inhibition of PI3-kinase results in enhanced levels of CHT proteins at the cell surface by decreasing their rate of internalization.

## Introduction

Forebrain cholinergic neurons control a range of physiological processes, including movement, cognition and attention [1,2], and are sensitive to pathology occurring in early AD [3,4]. A critical point in cholinergic transmission is the uptake of choline from the synaptic cleft by CHT to be used for synthesis of the neurotransmitter ACh [5,6]. Generally, choline uptake activity or binding of the CHT inhibitor hemicholinium-3 (HC-3) is found to be decreased in AD, or in mouse models of AD or neurons treated with β-amyloid peptides (Aβ) [7–11], although some studies have reported little or no changes in these measures [12–14].

The subcellular compartmentalization and trafficking of CHT proteins in neurons differs from that of some neurotransmitter transporters as CHT is not concentrated at the cell surface. A small pool of CHT proteins actively recycle between synaptic vesicles or endosomes and the cell surface, with the remainder located in intracellular pools that are possibly recruited to the plasma membrane by prolonged neuronal stimulation [15]. The density of CHT proteins at the plasma membrane can be increased by depolarization of the cell, thereby accelerating choline uptake. Thus, changes in cell surface CHT levels have an immediate and significant impact on choline uptake activity into cholinergic terminals and ACh synthesis. This may have functional parallels to the regulation of the insulin-sensitive glucose transporter (GLUT-4) in response to insulin signaling. GLUT-4 proteins are held in subcellular vesicles that move to the cell surface upon insulin stimulation [16].

Increasing evidence links changes in glucose metabolism, altered insulin receptor signaling, and insulin resistance and diabetes to an increased risk for the development of AD [17–20]. A rat model of type II diabetes reveals cortical neuron loss and increased soluble amyloid precursor protein (APP) and phospho-Tau [21]. Moreover, in APP_Swe_/PS1 AD-model mice, insulin resistance in brain can occur before changes in Aβ levels [22]. Importantly, a study of necropsy AD brains correlated increased Braak stage with decreased levels of insulin-like growth factor (IGF) signaling pathway components and decreased receptor binding affinity for insulin, IGF-1 and IGF-2 [23]. Another study noted decreased levels of insulin in cerebral cortex and IGF-I in hippocampus and hypothalamus of AD brains [24], and decreased Insulin Receptor Substrate 1 (IRS-1) responsiveness to insulin signaling in AD brains [25]. Interestingly, intranasal insulin administration improves cognition and decreases AD severity rating in humans [26,27] and rodent AD models [28].

Given the increases in both AD and insulin-resistant diabetes, and the link between declining cholinergic neuron function and the development of AD symptoms and pathology, it is important to understand functional changes in cholinergic neurons as a result of insulin signaling and resistance. This may inform the development of therapeutic strategies or highlight time-windows of beneficial intervention to maintain cholinergic neurons in healthy aging and pathology.

## Materials and Methods

### Materials

Insulin was purchased from Sigma-Aldrich (St. Louis, MO, USA). [methyl-^3^H]Choline chloride and [^3^H]HC-3 were from Perkin-Elmer (Waltham, MA, USA). 2-(4-morpholinyl)-8-phenyl-4H-1-benzopyran-4-one (LY294002) was from Millipore (Etobicoke, ON, Canada). All cell culture media and reagents were from Life Technologies (Burlington, ON, Canada). EZ-link sulfo-NHS-SS-biotin and Neutravidin agarose resin were from Thermo Fisher Scientific (Ottawa, ON, Canada). AlexaFluor-555 anti-Rb Zenon labeling kits and all fluorescent secondary antibodies were from Molecular Probes, Life Technologies (Burlington, ON, Canada). Polyclonal CHT antibody was prepared as described previously [29], anti-FLAG antibody was from Sigma-Aldrich (Oakville, ON, Canada), anti-actin antibody was from Santa Cruz Biotechnology (Santa Cruz, CA, USA), phospho-Akt and pan-Akt antibodies were from Cell Signaling Technology (Danvers, MA, USA) and horseradish peroxidase-conjugated goat anti-rabbit and goat anti-mouse secondary antibodies were from Jackson ImmunoResearch Laboratories (West Grove, PA, USA). Cell culture dishes were from VWR (Mississauga, ON, Canada) with the exception of glass-bottomed dishes supplied by MatTek Corp (Ashland, MA, USA) (0.16–0.19 mm coverslip thickness) used for TIRF imaging.

### Cell culture

The experiments were performed in SH-SY5Y human neuroblastoma cells that were stably transfected to express FLAG epitope-tagged rat CHT (called SY5Y-CHT cells), described previously [30]. Cells were maintained in Dulbecco’s modified Eagle’s medium (DMEM) containing 10% fetal bovine serum (FBS), 100 U/ml penicillin, 100 μg/ml streptomycin and 100 μg/ml geneticin (G418). Differentiation of wild-type SH-SY5Y and SY5Y-CHT cells was induced by adding 10 μM all-*trans*-retinoic acid at the time of plating for 3 days prior to assay or cell collection. At 24 h before assay/collection, the culture media was replaced with fresh retinoic-acid-containing media supplemented with either vehicle as a control or one of the following treatments: 20 nM insulin (vehicle is diluted acidified water) or 10 or 50 μM PI3-Kinase inhibitor LY294002 dissolved in dimethylsulfoxide (DMSO) (vehicle is DMSO).

### [^3^H]Choline uptake assay

SY5Y-CHT cells were plated on 35 mm dishes and treated as described in the text. Monolayers of cells were washed and incubated at 37°C in Krebs-Ringers-HEPES buffer (KRH) (124 mM NaCl, 5 mM KCl, 1.3 mM MgSO_4_, 1.5 mM CaCl_2_, 10 mM D-glucose, 20 mM HEPES-NaOH pH 7.4), with acute drug treatments added to yield the final concentrations indicated in the text. For the choline uptake assay, cells were incubated in 0.5 μM [^3^H]choline (0.5 μCi/ml) in the presence of absence of 1 μM HC-3 for 5 min, then placed on ice, washed twice with cold KRH and lysed in 0.1 M NaOH. Aliquots of these cell digests were analyzed for tritium content by liquid scintillation spectrometry using a Liquid Scintillation Counter (Packard-Perkin-Elmer Life Science, Boston, MA) and for protein content using a standard Bradford assay. In each experiment, each condition was assayed in triplicate and results were normalized to sample protein content. Specific [^3^H]choline uptake was calculated as the difference between total uptake in the absence of HC-3 and non-specific uptake in the presence of HC-3. To assess CHT transport kinetics, choline concentrations between 0.25 and 4 μM were used (specific activity constant at 0.2 Ci/mmol), with the uptake assay performed as described above.

### [^3^H]HC-3 binding assay

Monolayers of SY5Y-CHT cells plated on 6-well dishes were washed in KRH and treated with either vehicle or 20 nM insulin in KRH at 37°C for 5 min. Cells were then washed in cold KRH and placed on ice for 20 min to stop protein trafficking activity. Cells were incubated with [^3^H]HC-3 for 1 h on ice. Following incubation, cells were washed twice with KRH and digested using 0.1 M NaOH. Aliquots of cell digests were analyzed for tritium and protein content as described above. Each experiment was performed in triplicate and the results for each sample were normalized to sample protein content. Specific [^3^H]HC-3 binding was calculated as the difference between total binding measured in the absence of 1 μM HC-3 and non-specific HC-3 binding determined in the presence of 1 μM HC-3. To assess binding kinetics, [^3^H]HC-3 binding was measured over the range of 0.5–10 nM HC-3 with the specific activity of [^3^H]HC-3 held constant at 0.23 Ci/mmol.

### Cell surface biotinylation

Cells were plated on 60 mm dishes and treated as described in the text. Cells were then washed with HEPES-buffered saline solution (HBSS). For acute treatments, the drug was added to the HBSS to give the final concentrations indicated and cells incubated at 37°C for the described time period. After treatment, cells were washed in cold HBSS and incubated on ice to stop protein trafficking. Proteins at the cell surface were biotinylated on ice by incubating with 1 mg/ml sulfo-NHS-SS-biotin in HBSS for 1 h. Unbound biotin was quenched by washing and incubating cells in cold 100 mM glycine in HBSS. Cells were washed in HBSS and lysed at 4°C for 30 min in lysis buffer comprised of 1% Triton-X-100, 1% NP-40, 0.2% sodium-dodecyl sulphate, 150 mM NaCl, 50 mM Tris-HCl, pH 7.4, 1% protease inhibitor cocktail (Sigma), and 700 U/ml DNase1. Protein content was measured by Bradford assay, and an equal amount of protein from each sample applied to Neutravidin beads and the volumes normalized using lysis buffer. Samples and beads were rotated overnight at 4°C and non-bound proteins removed by 4 washes in cold lysis buffer. Bound protein was eluted by incubating for 10 min at 55°C in Laemmli sample buffer (2% SDS, 10% glycerol, 62.5 mM Tris-HCl, pH 6.8, 2.5% β-mercaptoethanol and 0.001% bromophenol blue) and CHT protein levels assessed by immunoblot.

### Immunoblotting

Cells were lysed in a lysis buffer comprised of 50 mM Tris-HCl pH 7.5, 150 mM sodium chloride, 1% Triton-X-100 and prepared in 3x Laemmli sample buffer. Following the separation of proteins on 7.5% SDS-PAGE gels, they were transferred to polyvinylidene difluoride (PVDF) membranes pre-wetted in methanol. Membranes were blocked in 10% non-fat milk powder dissolved in either phosphate-buffered saline containing 0.15% Trition-X-100 (rabbit anti-CHT) or Tris-buffered saline containing 0.1% Tween-20 (rabbit anti-actin, pan/phospho-PKB/Akt), then incubated overnight at 4°C with antibody in 5% milk powder in the appropriate buffer. After washes, membranes were incubated for 1–2 h at room temperature with peroxidase-conjugated goat anti-rabbit secondary antibody in 5% milk powder, then washed again. Immunoreactive proteins were detected using Enhanced ChemiLuminescence (Amersham ECL, GE Health Sciences, QC, Canada or Clarity ECL, BioRad, Mississauga, ON, Canada) and imaged with a ChemiDoc MP Imaging system (BioRad). Quantification was performed using the BioRad software ImageLab5.

### Total internal reflection microscopy (TIRF)

To fluorescently-label FLAG-CHT proteins in live SY5Y-CHT cells, cells were grown in glass-bottom dishes and treated in these as indicated. Cells were then washed twice in HBSS, and AlexaFluor 555-labeled rabbit anti-FLAG antibody was added to cells in HBSS at 37°C and incubated for 20 min. During this time, the fluorescent probe could bind to the extracellularly-located FLAG-tagged amino-terminus of CHT proteins and the CHT-antibody complex could undergo endocytosis into the cells. Following this incubation, cells were washed 3 times in HBSS to remove unbound fluorescently-labeled anti-FLAG antibody. For depolarization of cells by KCl addition during TIRF imaging, a pulled glass micropipette containing either 1 M or 2.5 M KCl (as indicated in the Fig legends) was attached to a FemtoJet microinjection system (Eppendorf, Mississauga, ON, Canada) and clamped in position above selected cells. The KCl solution was ejected at 150 psi over 0.1 seconds. Images were acquired using a Leica AM TIRF MC using an HCX PL APO 63X oil immersion objective with numerical aperture of 1.47. TIRF imaging experiments were carried out at room temperature to reduce the rate of cellular trafficking events.

### Data analysis

Data are presented as the mean ± SEM with *n* values showing the number of independent experiments performed on separate populations of cells. GraphPad Prism5 was used for data analysis and the assessment of statistical significance using paired or unpaired Student’s *t-*test or repeated measures ANOVA with post-hoc test, as appropriate [31]. Statistical significance was defined as *p* ≤ 0.05. Exponential analysis of fluorescence decay curves generated in the TIRF imaging experiments was performed using Origin Pro 9.1.

## Results

### Chronic Insulin Exposure Decreases CHT Activity and Suppresses PKB/Akt Activation

**Fig 1:**
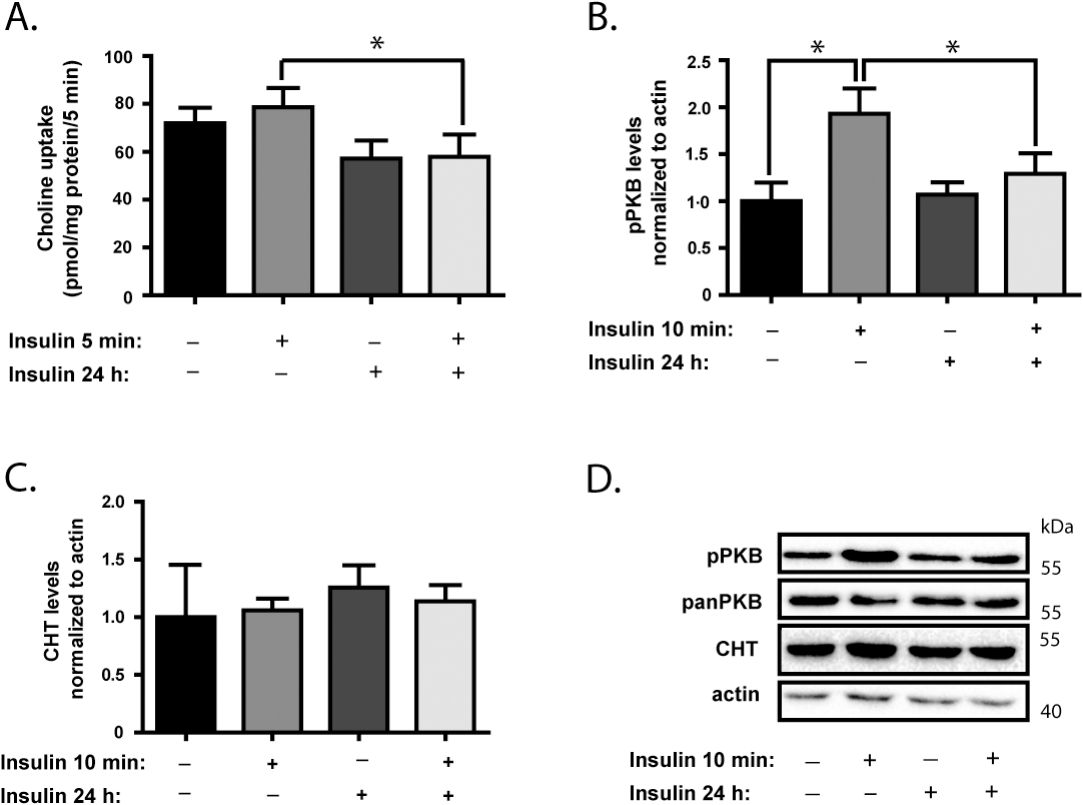
SH-SY5Y cells are responsive to acute insulin stimulation and altered insulin signaling impacts choline uptake by CHT. Differentiated SH-SY5Y cells stably expressing FLAG-tagged CHT proteins (SY5Y-CHT cells) were grown for 24 h in either control media or media containing 20 nM insulin, then either vehicle (water) or an additional 20 nM insulin was added acutely prior to analysis. **Panel A**. HC-3-sensitive choline uptake activity was determined as the difference between uptake in the absence minus the presence of 1 μM HC-3, and data are calculated as pmol / mg protein per 5 min for 7 independent experiments in this graph. There was negligible HC-3-sensitive choline uptake into non-transfected SH-SY5Y cells or cells that expressed the empty vector pcDNA3.1 (data not shown). **Panels B-D.** Following this treatment paradigm, cells were lysed for immunoblot analysis of the levels of phospho-PKB/Akt (Ser473), which is activated by insulin receptor signaling, pan-PKB/Akt, CHT protein and actin. The anti-CHT antibody used can detect both endogenous CHT and FLAG-tagged CHT proteins expressed in these cells. **Panel B.** Densitometric analysis of pPKB/Akt levels were normalized to sample actin levels for 9 independent experiments. **Panel C.** Densitometric analysis of CHT protein levels were normalized to sample actin levels for 8 independent experiments. **Panel D.** The representative immunoblots shown are from 5 independent experiments. Data in Panels A to C are expressed as the mean ± SEM, with statistically-significant differences assessed by repeated-measures ANOVA; asterisks denote *p* ≤ 0.05.

Our initial studies were designed to determine if either acute or chronic exposure of neural cells to insulin results in a change in choline uptake activity by CHT. The experimental model used was SH-SY5Y human neuroblastoma cells stably transfected to express FLAG-epitope tagged rat CHT protein (SY5Y-CHT cells), and differentiated by retinoic acid for 3 days to enhance the expression of neuronal and cholinergic neuronal phenotype markers [32–34]. Cells were either grown in control media for 24 h or media supplemented with 20 nM insulin, then acutely stimulated with 20 nM insulin or water (vehicle control) shortly before analysis. As illustrated in Fig 1A, acute addition of insulin to cells grown in control media did not significantly alter choline uptake activity. However, we did find that choline uptake activity by CHT is significantly higher in control cells that have been stimulated acutely by insulin when compared to cells grown for 24 h in insulin before acute insulin stimulation (*p* < 0.05) (Fig 1A).

Next, we tested the responsiveness of insulin receptor signaling pathways in these neural cells to the addition of insulin or treatment with an inhibitor of this signaling process. We demonstrate that, when grown in control culture medium, SY5Y-CHT cells respond to the acute addition of 20 nM insulin with a significant increase in phosphorylation of PKB/Akt, a key downstream target activated in the insulin receptor signaling pathway (Fig 1B and 1D). However, when the culture media is supplemented with 20 nM insulin for 24 h prior to testing, the response of cells to acute exposure to a further 20 nM of insulin is significantly attenuated and does not result in increased phosphorylation of PKB/Akt, suggesting that prolonged insulin treatment creates an insulin resistant state. There is no change observed in the levels of CHT protein during the course of these treatments (Fig 1C and 1D).

**Fig 2:**
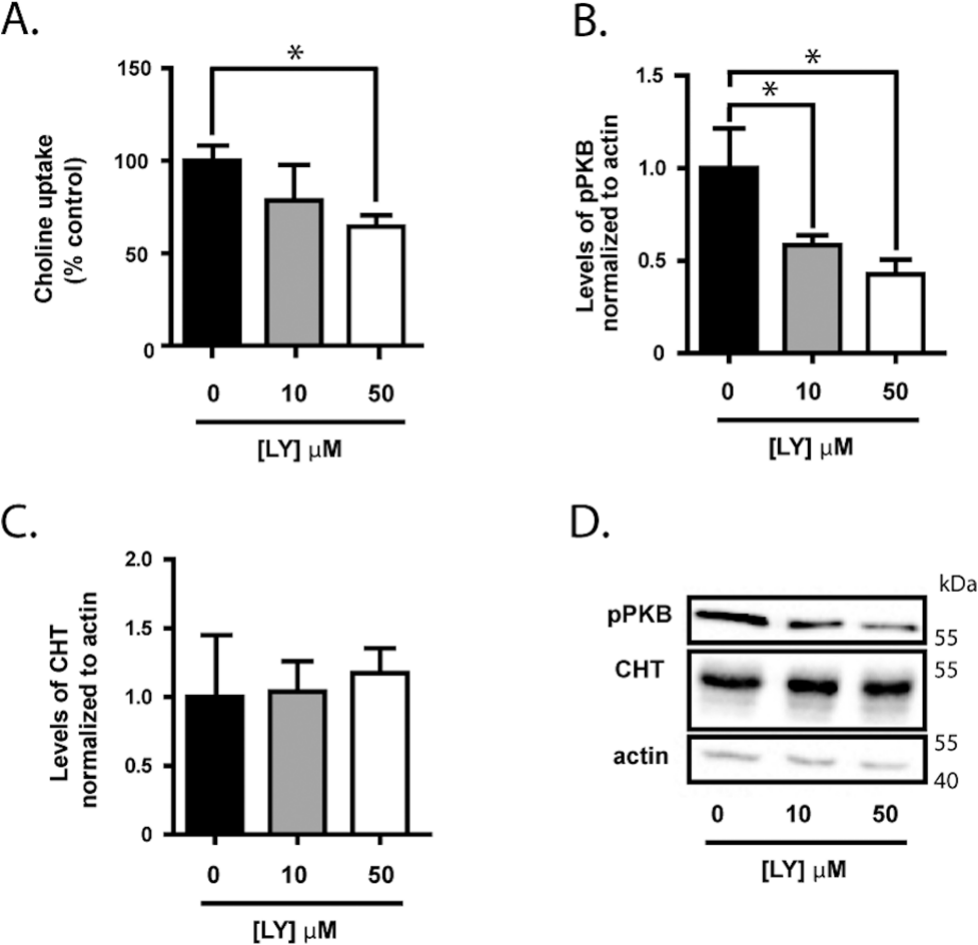
Inhibition of PI3-Kinase alters choline uptake in SH-SY5Y cells. Cells were treated with the PI3-Kinase inhibitor LY294002 with the concentrations indicated in the Fig **Panel A.** HC-3-sensitive choline uptake by CHT was measured as indicated in Fig 1 following 5 min exposure of differentiated SY5T-CHT cells to LY294002 treatment. Data are normalized to the control treatment group for n = 3 independent experiments. **Panels B-D.** Cells were prepared for immunoblot analysis of pPKB/Akt, CHT and actin levels following 5 min treatment with LY294002 from 4 independent experiments. **Panel B.** Densitometric analysis of pPKB levels were normalized to sample actin levels. **Panel C.** Densitometric analysis of CHT protein levels were normalized to sample actin levels. **Panel D.** The representative immunoblots shown are from 4 independent experiments. Data in Panels A to C are expressed as the mean ± SEM, with statistically-significant differences assessed by repeated-measures ANOVA with asterisks denoting *p* < 0.05.

Conversely, when the PI3-Kinase pathway inhibitor LY294002 is applied to cells acutely (5 min pre-incubation), choline uptake activity by CHT was significantly decreased by 50 μM, but not by 10 μM, of the drug (Fig 2A). As predicted, treatment of these cells with either 10 μM or 50 μM LY294002 resulted in significantly decreased phosphorylation of PKB/Akt (*p* < 0.05) (Fig 2B and 2D). Total CHT protein levels are not significantly changed by treatment with LY294002 (Fig 2C and 2D).

### The Dynamics of CHT Movement to and from the Cell Surface are Regulated by Insulin Signaling

**Fig 3:**
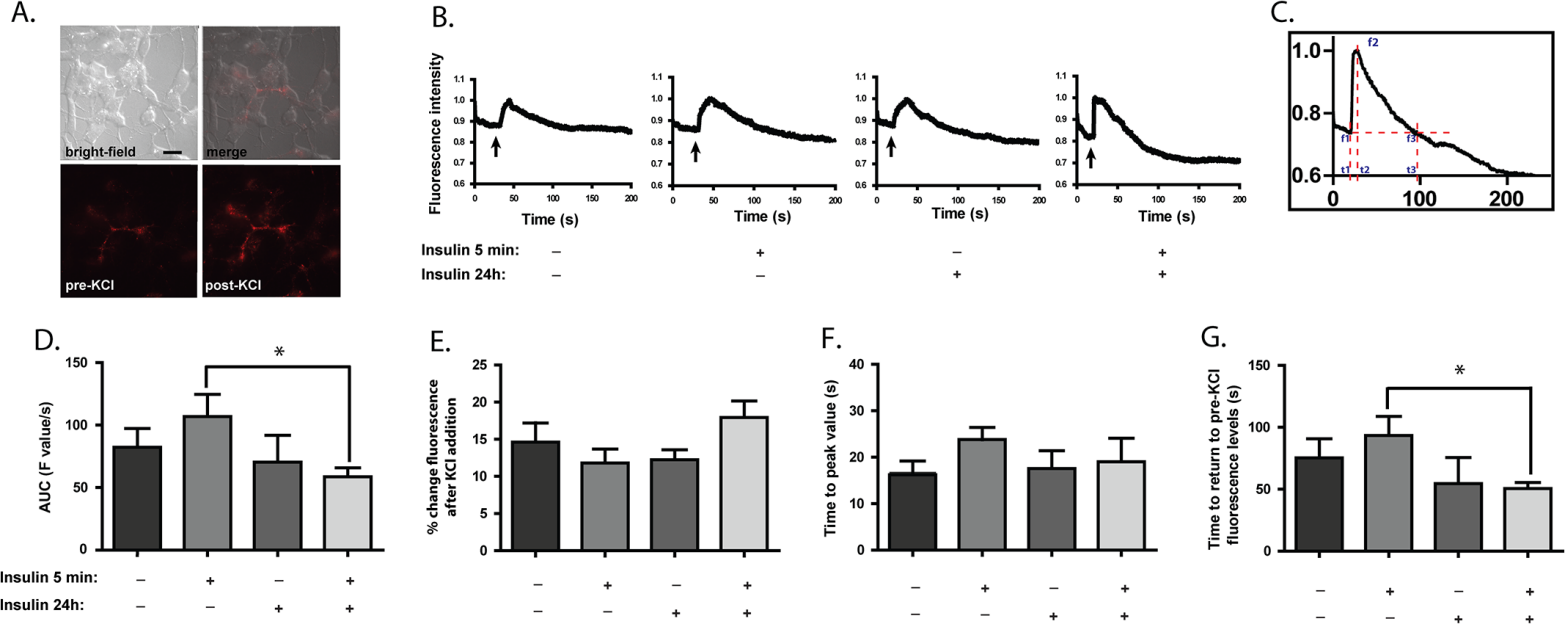
Dynamics of CHT internalization are altered by acute insulin treatment. Cells were grown for 24 h with the addition of either vehicle (water) or 20 nM insulin. To facilitate TIRF microscopy, FLAG-CHT was labeled by 20 min incubation at 37°C with AlexaFluor 555-labeled rabbit anti-FLAG antibody, then the cells were washed 3 times with HBSS at the end of this period to remove background non-specific labeling. The initial experiments tested the ability of either acute vehicle or insulin addition to alter total cell fluorescence levels related to the movement of CHT proteins to the cell surface; neither of these treatments caused significant changes in fluorescence levels. **Panel A.** Bright-field and fluorescence images of cells prior to and after KCl addition with scale bar indicating 20 μm. The imaging protocol was as follows: the culture dish was set on the stage of the TIRF microscope, either insulin or vehicle were added and imaging was started for 30–60 sec, then a small volume of 1 M KCl was applied to the area of the cells by pressure ejection and imaging was continued for a further 3–4 min. Images were captured at 150 nm depth from the coverslip, and experiments were performed at room temperature to reduce the rate of cellular trafficking events being imaged. **Panel B.** Representative traces of changes in the cellular fluorescence levels over the time course of imaging with various treatments, as indicated. The arrows indicate when KCl was applied to the cells. **Panel C.** Schematic representation indicating analysis of changes in cell fluorescence in live cells in **Panels D – G. Panel D.** Area-under-the-curve (AUC) analysis of fluorescence images calculated as the sum of fluorescence values between t1 and t3, analyzed using GraphPad Prism v.5.0. ***Panel E*.** Percentage change in cell surface fluorescence levels following K^+^-depolarization levels, calculated using the formula [(f2-f1)/((f1+f2)/2)*100)]. **Panel F.** Time-to-peak value calculated as the time at point t2 minus that at point t1. **Panel G.** Time taken for cell fluorescence to return to baseline values calculated as time point t3 minus time point t2. Typically 2 or 3 regions of interest having high fluorescence corresponding to 2 to 3 individual cells were chosen for analysis per culture dish, with 4 to 5 dishes analyzed per treatment group in 2 or 3 independent experiments. Data in Panels D to G are expressed as the mean ± SEM, with statistically-significant differences assessed by unpaired Student’s *t*-test; asterisks denote *p* ≤ 0.05 compared to cells grown in control conditions (absence of 24 h treatment with insulin) and acutely stimulated with insulin.

Choline uptake activity by CHT is strongly linked to neuronal activity and neurotransmitter release, and the impact of changes in cellular signaling on this process may only be fully assessed with the examination of depolarized or stimulated neuronal cells. CHT protein dynamics and regulation have been studied in resting neurons, but much less is known about its regulation in stimulated neurons and that information comes only from analysis using biochemical methods. The trafficking of CHT proteins in the actively recycling pool to and from the cell surface is an important determinant of the plasma membrane level of CHT proteins and the ability of cholinergic neurons to reclaim choline from the synaptic cleft for ACh synthesis [15]. To investigate this, we utilized TIRF microscopy of live SH-SY5Y cells expressing FLAG-tagged CHT proteins in conjunction with fluorescently-labeled anti-FLAG antibodies. This permits the excitation of the fluorescent probe (i.e. antibody) at very limited distances from the cell surface (maximum 150 nm from the coverslip), thereby allowing us to assess the movement of CHT proteins to and from the plasma membrane and at the cell surface in live cells. These cells were depolarized by delivery of small volumes of KCl into the media by pressure ejection from a glass pipette (microelectrode) positioned above the cells at a defined point during imaging. Representative TIRF images of cells expressing FLAG-tagged CHT with fluorescently-labeled anti-FLAG antibody attached are shown in Fig 3A before and after KCl stimulation.

**Fig 4:**
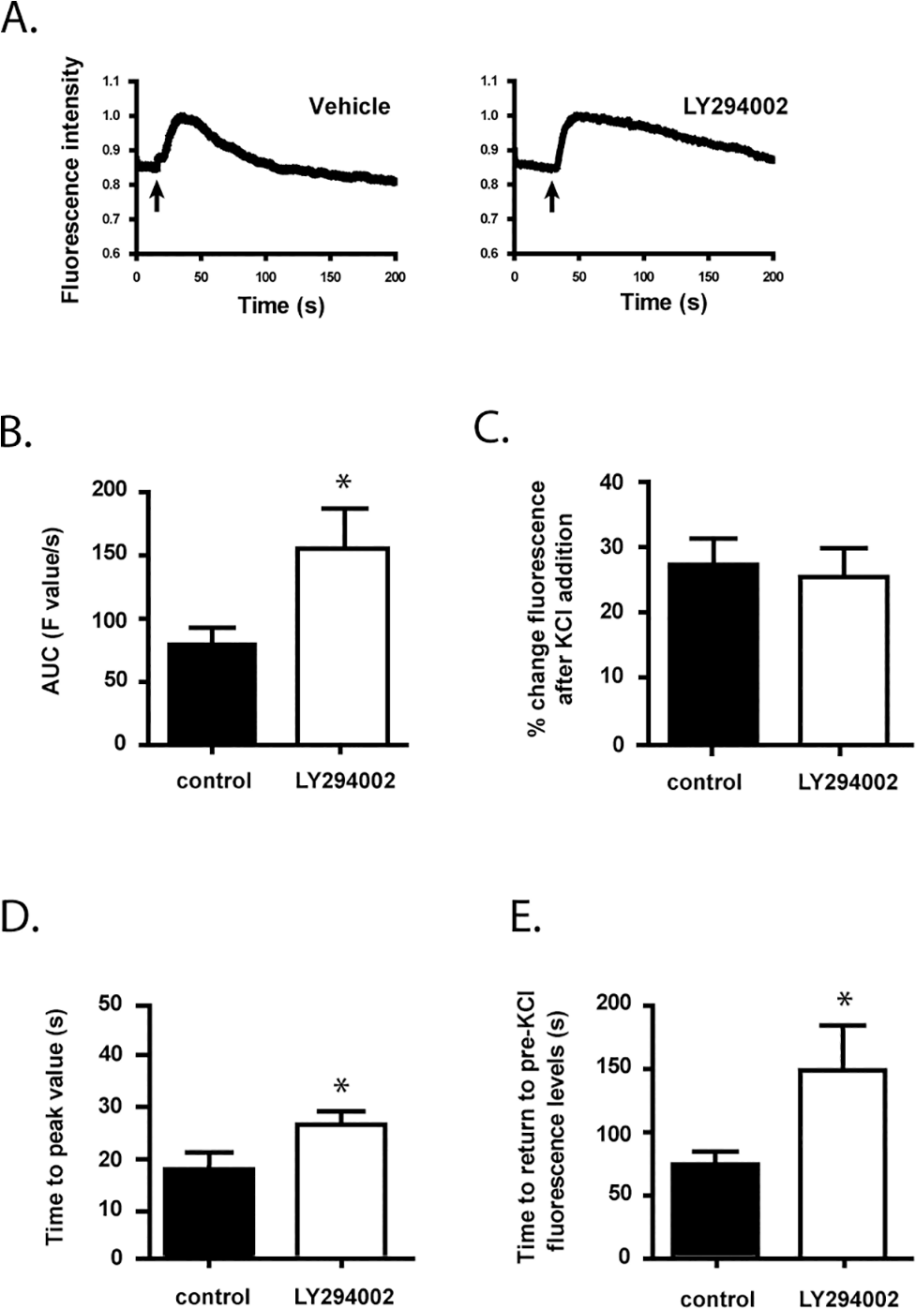
Dynamics of CHT trafficking are altered by LY294002 treatment. Cells were grown for 24 h in the presence of either vehicle or 10 μM LY294002 before being imaged by TIRF microscopy. CHT proteins were fluorescently labeled as in Fig 3. The imaging protocol was as follows: the dish was set on the stage of the TIRF microscope and imaging was started for 30–60 sec, then a small volume of 2.5 M KCl was applied to the area of the cells by pressure ejection and imaging was continued for a further 3–4 min. Images were captured at 150 nm depth from the coverslip, and experiments were performed at room temperature. **Panel A.** Representative traces of changes in the cellular fluorescence levels over the time course of imaging with various treatments, as indicated. The arrows indicate when KCl was applied to the cells. **Panel B.** Area-under-the-curve (AUC) analysis of fluorescence images was calculated as described in Fig 3. **Panel C.** Percentage changes in cell surface fluorescence levels following K^+^-depolarization levels calculated as described in Fig 3. **Panel D.** Time-to-peak value calculated as described in Fig 3. **Panel E.** Time taken for cell fluorescence to return to baseline values calculated as described in Fig 3. In these experiments, 1 to 5 regions of interest having high fluorescence corresponding to 1 to 5 individual cells were analyzed per culture dish, with 4 to 5 dishes analyzed per treatment group in 3 independent experiments. Data in Panels B to E are expressed as the mean ± SEM, with statistically-significant differences assessed by unpaired Student’s *t*-test; asterisks denote *p* ≤ 0.05.

In these experiments, cells were imaged initially for 30–60 sec to establish the baseline for individual cell CHT fluorescence, and then cells were depolarized by KCl. Changes in the level of fluorescently-tagged CHT proteins close to the plasma membrane were monitored for a further 3–4 min with images captured at 150–200 ms intervals. Representative examples of the changes in individual cell fluorescence intensity over time that are generated with the different treatments are shown in Fig 3B. In all treatment groups, K^+^-addition induced a sharp rise in cell surface fluorescence levels and this then decayed over time to baseline levels. The key values taken for analysis of these data are indicated in Fig 3C, a cropped image highlighting a K^+^-mediated spike in fluorescence levels. From the data generated for changes in fluorescence intensity over time, we selected the values from the point when fluorescence begins to rise (t1, f1) until the point when it returns to the pre-stimulation intensity level (t3, f3). These values were used to determine the area-under-curve (AUC), time from t1 to peak value, time from peak value to t3 and percentage rise in height (see legend). For all treatment groups assessed in Figs 3 and 4, further analysis of the shapes of the fluorescence decay curves between the peak values and the return to baseline values revealed that they are fit best by a single exponential function.

We used the experimental paradigm from Fig 1 in cells that now undergo K^+^-induced depolarization to test the impact of depolarization on the effects that acute and longer-term insulin exposure may have on CHT dynamics. Our initial experiments determined that acute exposure of non-depolarized cells to insulin caused only a small, transient increase in cellular fluorescence levels, but this did not differ significantly from the signal generated with vehicle (water) in terms of the length of time that the fluorescence levels were altered (data not shown).

We find that when cells are stimulated acutely by insulin just prior to the application of K^+^, the AUC (sum of fluorescence levels between t1 and t3) for changes in fluorescence associated with CHT protein at the cell surface was slightly increased relative to that measured in control cells, although this did not achieve statistical significance. A critical observation is that longer-term (24 h) exposure of cells to insulin reduces the AUC measurement for cell surface CHT fluorescence mediated by acute insulin addition (Fig 3D), resulting in a significant difference in the response of cells cultured for 24 h in insulin prior to insulin stimulation when compared to cells cultured in control media prior to stimulation (*p* < 0.05). This matches our findings with the attenuating effect of prolonged insulin exposure on acute insulin treatment on choline uptake activity in non-depolarized cells shown in Fig 1A. Breaking this down into the individual components of the data captured by TIRF imaging, we find that there is not an alteration in either the maximal cell surface fluorescence levels achieved (peak amplitude) related to CHT protein movement to the plasma membrane (Fig 3E) or in the time taken to reach the peak amplitude of fluorescence (Fig 3F) with any of the treatments tested relative to the changes in control vehicle-treated cells. Finally, there is a significant decrease in the time taken to return to baseline levels of fluorescence between cells cultured for 24 h in insulin and cells cultured in control media before acute insulin stimulation (Fig 3G) thereby contributing to the significant difference between cells cultured in these conditions (*p* < 0.05).

Next, we investigated the effect of exposure of cells to the PI3-Kinase inhibitor LY294002 on CHT protein dynamics with K^+^-mediated depolarization. Representative traces of changes in fluorescence associated with CHT protein levels at the cell surface are shown in Fig 4A. Again, we quantified the AUC as a measure of the total fluorescence generated by the movement of CHT proteins to the cell surface and the length of time that they reside there. The 10 μM LY294002 treatment results in a significantly greater AUC when compared to control cells (Fig 4B) (*p* < 0.05). While the peak amplitude of response is unchanged for LY294002 treatment relative to control (Fig 4C), both the times to reach peak values (Fig 4D) and to return to baseline levels are significantly longer (Fig 4E) when compared to control cells (*p* < 0.05).

### The Cell Surface Level of CHT is not Altered by Insulin in Resting Cells

**Fig 5:**
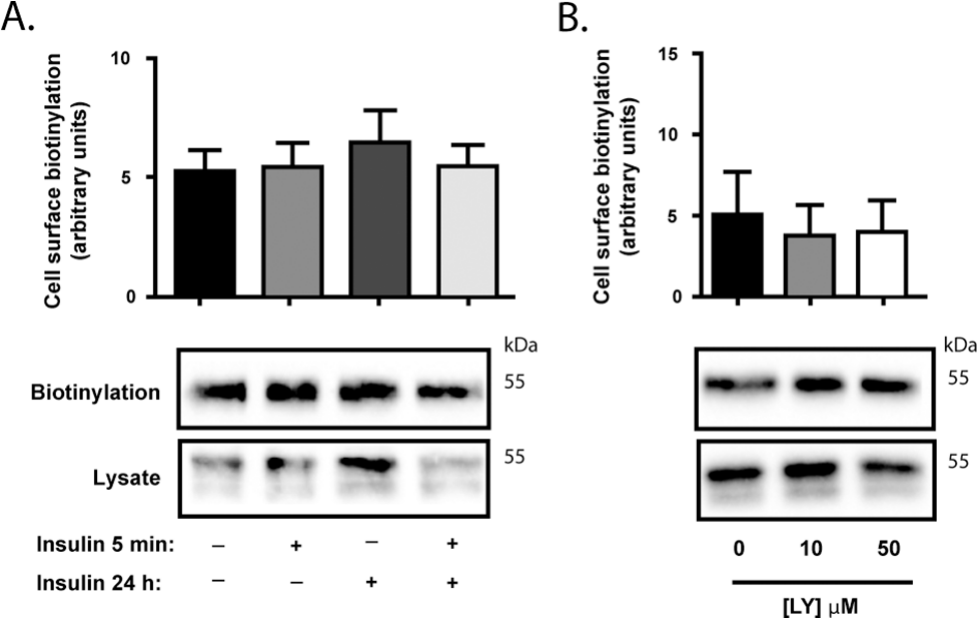
Cell surface CHT levels are unchanged by altered PI3-Kinase signaling in non-depolarized cells. Groups of SY5Y-CHT cells were treated with either vehicle or 20 nM insulin for 24 h, then prior to analyzing cell surface CHT levels cells were treated for 5 min with either vehicle, 20 nM insulin or 10 μM LY294002 as indicated. Cells were then cooled to 4°C and biotin added to cells on ice for 1 h before washing and lysing cells. **Panel A.** Densitometric analysis of immunoblots of biotinylated CHT recovered from cell surface protein biotinylation assays of vehicle or insulin-treated cells were normalized to CHT protein content in total cell lysates. Representative immunoblots for biotinylated CHT and cell lysate samples are shown, with 8 independent experiments analyzed. **Panel B.** Densitometric analysis of immunoblots of biotinylated CHT recovered from cell surface protein biotinylation assays of vehicle or LY294002-treated cells were normalized to CHT protein content in total cell lysates. Representative immunoblots for biotinylated CHT and cell lysate samples are shown, with 3 independent experiments analyzed. Data are expressed as the mean ± SEM for densitometry, with arbitrary units representing a measure of immunoblot band intensity.

Under resting (non-depolarized) conditions, only a small proportion of the total cellular CHT proteins are present at the cell surface, with the remainder divided between a small actively recycling pool and an intracellular store that may release CHT proteins to the recycling pool during prolonged neuronal stimulation [15]. One mechanism by which a perturbation of the neurons may alter choline uptake activity is through a redistribution of more CHT proteins to the cell surface. Thus, we used labeling of cell surface proteins with biotin to evaluate changes in CHT protein disposition in cells treated with either insulin or the PI3-kinase inhibitor. As shown in Fig 5, the level of cell surface CHT proteins is not significantly altered by either insulin (Fig 5A) or LY294002 (Fig 5B) treatment. This was found with either acute or longer-term insulin treatment or with the inhibition of PI3-Kinase signaling. This suggests that in non-depolarized neural cells alterations in insulin signaling do not lead to changes in CHT cell surface levels that were measurable with this approach.

We addressed this also by measuring the kinetic parameters for function of CHT in control and insulin-treated neural cells. First, steady-state binding assays using the CHT antagonist [^3^H]HC-3 over a range of concentrations from 0.5 to 10 nM were performed at 4°C to prevent the internalization and recycling of proteins from the cell surface. Based on the evaluation by Michaelis-Menton kinetics to calculate the binding affinity (K_D_) and maximum number of binding sites (B_max_) values for [^3^H]HC-3 binding to CHT proteins, we observed that neither of these kinetic parameters are altered by either acute or longer-term insulin treatment of cells (Table 1), in agreement with the lack of change in biotinylated cell surface CHT levels in resting state cells (Fig 5A). Second, we measured the kinetics of choline uptake, also over a concentration range thereby allowing the maximal rate of uptake (V_max_) and solute binding affinity (K_M_) to be determined. Again, there were no differences in these values for cells treated for 24 h with insulin when compared to control cells (Table 1).

**Table 1:** Insulin treatment, acute or chronic, does not alter the number of CHT proteins at the cell surface, their binding affinity or the maximal uptake of choline. Choline uptake and HC-3 binding were assessed over a concentration range to determine kinetic parameters. Data were fit by Michaelis-Menten nonlinear regression and are expressed as mean ± SEM of 5 experiments (choline uptake) and 3 experiments (HC-3 binding). Units: Choline uptake V_max_ = pmol / mg protein / 5 min, K_m_ = μM; HC-3 binding B_max_ = fmol / mg protein, K_D_ = nM. ND–not determined.

Treatment	V_max_ ± SEM [Choline uptake]	K_m_ ± SEM [Choline uptake]	B_max_ ± SEM [HC-3 binding]	K_D_ ± SEM [HC-3 binding]
Vehicle [24 h], then vehicle [5 min]	576.4 ± 134.1	0.70 ± 0.57	108.5 ± 21.8	0.16 ± 0.29
Vehicle [24 h], then insulin [5 min]	ND	ND	126.3 ± 19.8	0.17 ± 0.24
Insulin [24 h], then vehicle [5 min]	533.6 ± 102.2	0.31 ± 0.25	107.0 ± 17.6	0.27 ± 0.30
Insulin [24 h], then insulin [5 min]	ND	ND	139.0 ± 28.1	0.40 ± 0.42

## Discussion

We made several novel observations in the present study in relation to the response of CHT proteins to insulin treatment of neural cells. We demonstrate the effects of both acute and chronic insulin treatment, and the impact of inhibiting PI3-Kinase signaling on choline uptake in SH-SY5Y neuroblastoma cells that are transfected to stably express CHT (SY5Y-CHT cells). We show by TIRF microscopy imaging the dynamic movement of CHT proteins in live cells and the effects of acute and chronic drug treatments on the response of these proteins to K^+^-mediated depolarization.

First, we show that differentiated SY5Y-CHT cells respond to acute exposure of insulin with enhanced phospho-PKB/Akt levels, but that this is attenuated with long-term exposure of the cells to insulin. We did not determine the underlying mechanism, for example whether this is through saturation of insulin receptors in cells grown in hyper-insulinemic conditions or through down-regulation of insulin signaling cascade components, but the outcome is the inability of cells to respond to acute insulin stimulation thus serving as a model for cellular insulin resistance. We also show that choline uptake activity is significantly higher in cells grown in control conditions prior to acute insulin stimulation in comparison to cells that have been grown in raised insulin before stimulation. It has been shown previously that protein kinase C (PKC) [30,35] and protein kinase A (PKA) [36] signaling can regulate CHT activity and that treatment with phosphatase inhibitors can modulate the affinity of CHT for choline [37,38]. Modulation of the PI3-Kinase signaling pathway can control cell surface levels and activity of the dopamine transporter DAT [39,40], and both the serotonin and glutamate transporters are regulated by PKC [41,42]. Neuronal insulin resistance can lead to losses in synaptic plasticity, altered synaptic structure, and genes associated with neurotransmission are differentially expressed in a rat model of type II diabetes when compared to non-diabetic controls [43–45].

The hypothesis that brain insulin resistance may contribute to the development of AD pathology is receiving increasing attention [17–20]. Epidemiological studies document a link between type II diabetes and increased risk of developing AD [46–48], and brain insulin resistance has been described in patients with mild cognitive impairment or AD [23–25] and in animal models of AD [22]. Although the mechanisms underlying this relationship is not clear, it has been shown that Aβ can compete with insulin for binding to insulin receptors and alter insulin receptor signaling processes [49,50]. Future studies should be directed towards elucidating these mechanisms.

We also investigated the effect of inhibiting PI3-Kinase signaling using the inhibitor LY294002, and found that LY294002 treatment of cells impacts choline uptake activity. Acute treatment with a low concentration of inhibitor (10 μM), although sufficient to significantly decrease phospho-PKB/Akt activation, did not alter choline uptake. However, CHT activity is decreased significantly by a higher concentration of this inhibitor (50 μM). Since PI3-Kinase activity is necessary for neuronal survival [51], it must be considered that this may also relate to generalized dysregulation of cellular processes. In a study examining the effect of PI3-Kinase on CHT gene expression it was found that treating cells for 24 h with 10 μM LY294002, does not substantially alter CHT mRNA levels [52]; these investigators did not assess the effect of LY294002 on either CHT protein or choline uptake levels. In other studies, it was observed that treatment of PC12 cells or cultured rat septal neurons for 72 h with 10 μM LY294002 had no effect on basal ACh production, although it did reduce an NGF-mediated enhancement of ACh synthesis [53]. As choline uptake activity and ACh synthesis are closely coupled, this suggests that inhibition of PI3-kinase did not alter CHT function under basal conditions.

To gain a better understanding of the real-time dynamics of CHT trafficking, we used TIRF microscopy that is suited for rapid imaging of events occurring at or close to the cell surface. Neural cells were depolarized by KCl released by pressure ejection from a glass micropipette positioned adjacent to cells, and changes due to the amount of fluorescence associated with CHT proteins moving to and from the cell surface were imaged continuously. Thus, we could analyze how the components that characterize the response of CHT [*time to move to the cell surface*, *time remaining at the cell surface*, *and peak amplitude of CHT proteins at the surface*] are differentially regulated by drug treatments. The PI3-kinase inhibitor LY294002 significantly extends the time course for CHT proteins at the cell surface, suggesting that this signaling pathway may be important for or have a role in regulating CHT endocytosis; this drug also significantly increased the time for CHT movement to the cell surface, and together these led to a greater AUC value for the depolarization-induced response of CHT proteins. Our cell surface biotinylation experiments did not show an increased level of CHT protein at the plasma membrane of LY294002-treated cells compared to control, but this could relate either to lower sensitivity of this assay or the fact that the cells assessed in the biotinylation experiments were not depolarized. Further refinement of our understanding in the signaling events downstream of PI3-Kinase leading to this change may benefit our understanding of the mechanisms regulating CHT cell surface levels.

Chronic insulin treatment did not significantly change CHT peak values or times of exo- or endocytosis of CHT proteins, but a critical difference is seen when insulin is added to cells just prior to depolarization. Here an enhanced level of CHT proteins at the cell surface is found in cells acutely treated with insulin, but not in cells that were grown for 24 h in insulin prior to acute insulin challenge (insulin-resistant cells). Further examination of this reduced ability of CHT proteins to respond to insulin may improve our understanding of the underlying causes of cognitive impairment observed in AD patients with brain insulin resistance or provide insight into the therapeutic benefit found with intranasal insulin administration to AD patients [26,27].

In summary, we show that CHT activity or function can be regulated by modulating insulin receptor signaling, identifying a new target for the action of insulin in brain. We demonstrate the real-time dynamics of CHT movement to the cell surface following depolarization and show that this is altered by both transient and chronic stimulation and by the inhibition of insulin receptor signaling. This ability to dissect the different effects of each drug treatment on the mechanisms that regulate CHT cell surface levels may be beneficial in gaining a further understanding of cholinergic neuron responses to changes in the brain microenvironment.
